# Care for dementia patients and caregivers amid COVID-19 pandemic

**DOI:** 10.1016/j.cccb.2022.100040

**Published:** 2022-01-18

**Authors:** Avanthi Paplikar, Jayeeta Rajagopalan, Suvarna Alladi

**Affiliations:** a Dr. S.R.Chandrasekhar Institute of Speech and Hearing, Bengaluru, India, 560084; b Department of Neurology, National Institute of Mental Health and Neurosciences [NIMHANS], Bengaluru, India, 560029

**Keywords:** Dementia care, Caregiver support, COVID-19, Pandemic, Care guidelines, Recommendations

## Abstract

**Background:**

Multiple efforts have been taken across the world to bridge gaps in evidence and provide recommendations for dementia care and caregiver support during the COVID-19 pandemic. We aimed to review the available literature on measures used to alleviate the negative impacts of the pandemic on dementia care.

**Methods:**

We searched three databases: PubMed, CINAHL and PsycINFO to identify studies that described strategies that were recommended or taken to provide care and support to persons with dementia and their caregivers during the COVID-19 pandemic.

**Results:**

We included a total of 37 papers, originating from high, middle and low-income countries. We also included 6 papers exploring safety of COVID-19 vaccinations. Infection prevention recommendations predominantly involved providing consistent reminders to persons with dementia to engage in infection prevention measures and included measures to restrict wandering in long-term care settings to reduce spread of infection. Medical care included tele and video consultations to maintain and monitor clinical stability. Guidance for continuing rehabilitation activities for persons with dementia through remotely conducted cognitive stimulating activities, physical exercises and environmental modifications were further highlighted by multiple studies/articles. In addition, strategies to support caregivers were also advised and included teleconsultations and psycho-educational programs.

**Conclusion:**

The COVID-19 pandemic has exposed gaps in health and social care systems across the world, but has also revealed the enduring dedication of clinicians, expert groups and dementia support organizations to modify and adapt existing dementia care services to meet the needs of persons with dementia and their caregivers during periods of emergency.

## Highlights

• The dementia community is reframing existing care services during the pandemic.
• Persons with dementia must be reminded of infection prevention measures.
• Tele-consultations are feasible strategies to monitor persons with dementia.
• Functional rehabilitation activities should be continued and conducted remotely.
• Support caregivers through psycho-educational programs and tele-consultations.

## 1 Introduction

The World Health Organization (WHO) declared the COVID-19 pandemic as a public health emergency of international concern, affecting many countries, disproportionately claiming lives of the elderly [1] and posing significant challenges particularly for caregivers of those with impaired cognition [2,3].

### 1.1 Risk of infection in persons with dementia

The inherent cognitive deficits of individuals with dementia impairs their ability to comprehend, execute, and remember the multiple guidelines developed by public health authorities such as wearing masks, physical distancing, hand hygiene, covering mouth and nose while sneezing/coughing and monitoring or reporting COVID-19 symptoms [4], [5], [6].

Higher mortality and morbidity rates are reported in persons with dementia due to associated comorbidities compared to other individuals without dementia [4,5,7,8]. There are also reports suggesting an association between ApoE e4 genotype and severe COVID-19 infections [9,10] which could further explain the higher prevalence of COVID-19 in dementia patients.

### 1.2 Impact of clinical diagnosis and medical management in persons with dementia

Due to increase in COVID-19 cases, there was closure of non-essential services which included diagnostic and follow-up services, pharmacologic and non-pharmacologic management (occupational therapy, physical therapy, counselling, speech therapy) of dementia patients [5]. This affected essential visits for laboratory tests and neuroimaging [5,6] and contributed to delays in the diagnosis of new onset dementia and monitoring of rapidly progressive dementias as well [11]. However, as of 2021, multiple vaccines have been approved and administered for use for COVID-19 [12]. Although evidence on the safety of these vaccines in vulnerable groups such people with comorbidities is limited.

### 1.3 Impact of confinement on cognition and behavior of persons with dementia

Multiple studies have examined the impact of confinement measures associated with the COVID-19 pandemic on persons with dementia and their caregivers. Worsening or new onset of neuropsychiatric symptoms [13], [14], [15], [16], [17], [18] were found as an unintended consequence of pandemic induced measures, with agitation [14,16,18], depression [14,17], apathy [16,18] and anxiety [14] most commonly reported in persons with dementia. In addition, cognitive decline in persons with dementia was also observed. A study in Portugal [13] revealed that 80% of caregivers perceived a worsening in cognition among their care recipients as a result of home confinement. Similar findings were also reported by other European studies [14,16]. These changes in behaviors and cognition in persons with dementia have further exacerbated caregiver distress [13], [14], [15], [16], [17]. These findings demonstrate that the restrictions imposed to contain the pandemic have had significant negative consequences for the cognitive and psychological well-being of persons with dementia [19] and their caregivers.

### 1.4 Impact on caregivers of persons with dementia

Significant disruptions to daily routines due to the pandemic led to numerous challenges in caring for persons with dementia and reduced supports available to caregivers, which in turn contributed to caregiver burnout and stress. Altieri and Santangelo [3] reported increased levels of depression and anxiety in caregivers of persons with dementia due to the COVID-19 pandemic and lockdown restrictions that were induced as a result. The restrictions amplified the daily challenges experienced by caregivers, which precipitated feelings of loneliness, social isolation and stress because of social distancing [20]. As a result of COVID-19 pandemic, many caregivers found themselves providing more care than they used to previously and were likely to be in fair-to-poor health as a result of balancing high levels of household work and challenges faced due to coordinating care with healthcare professionals [21].

In response to the challenges encountered by persons with dementia and their caregivers during the COVID-19 pandemic, in the initial phase of the pandemic, several expert groups developed recommendations to guide care of persons with dementia and ensure their well-being and safety. Given the wide range of care strategies that were developed, there is a need to review different approaches and consolidate the various efforts made to mitigate the negative consequences of the pandemic. This effort will aid in developing strong evidence-based guidelines that can inform dementia care practice during the ongoing COVID-19 pandemic.

In this paper, our objective was to review available studies and methods that aimed to alleviate the wide range of negative impacts of COVID-19 pandemic on persons with dementia and their caregivers.

## 2 Methods

We reviewed literature regarding the provision of dementia care during the COVID-19 pandemic across the world, with specific focus towards measures or recommendations for dementia care and support for caregivers.

### 2.1 Selection of studies

We included studies that described measures that were taken or could be taken to provide daily care, medical care or general support to persons with dementia or cognitive impairment and their caregivers. The inclusion criteria included: 1) Articles by experts and expert groups that described strategies or recommendations to provide dementia care during the COVID-19 pandemic; 2) Primary research studies, special articles, perspective articles, reports, commentaries, recommendations and guidelines; and 3) Articles and studies that are peer-reviewed.

We excluded articles that were not in English, reviews, papers not specific to persons with dementia or cognitive impairment, those which focused only on the impact of confinement on persons with dementia and their caregivers and any grey literature.

### 2.2 Search strategy

We performed the literature searches in April 2021, with the last search conducted on April 12^th^, 2021. We searched three main databases: PubMed, CINAHL and PsycINFO. A search strategy was initially developed for PubMed and later translated for CINAHL and PsycINFO using the database specific search syntax. In order to identify key subjects and search terms we identified search terms used in other reviews [19,22] and screened the literature available on dementia care and COVID-19 through Google Scholar. The search strategy focused on title, abstract and keyword searches across all three databases. The complete search strategy for all databases is provided in Appendix 1. A similar search strategy was further adopted on October 29^th^, with the aim of including studies on vaccinations. The complete search strategy for vaccinations for all databases is provided in Appendix 2. We also searched Google Scholar using the search terms ‘vaccination’, ‘COVID 19’ and ‘dementia’.

The results were exported to Mendeley, where they were de-duplicated. They were also exported in excel format from each of the individual databases. The title, abstract and full texts were screened by two reviewers [JR and AP].

### 2.3 Data extraction and synthesis

A template was formulated to extract data and included information on author, publication country, type of article, sample size, setting, daily care, medical care, social care and support for caregivers. Two authors [JR and AP] completed this data extraction.

## 3 Recommendations for dementia care and caregiver support during the COVID-19 pandemic

**Fig. 1.**
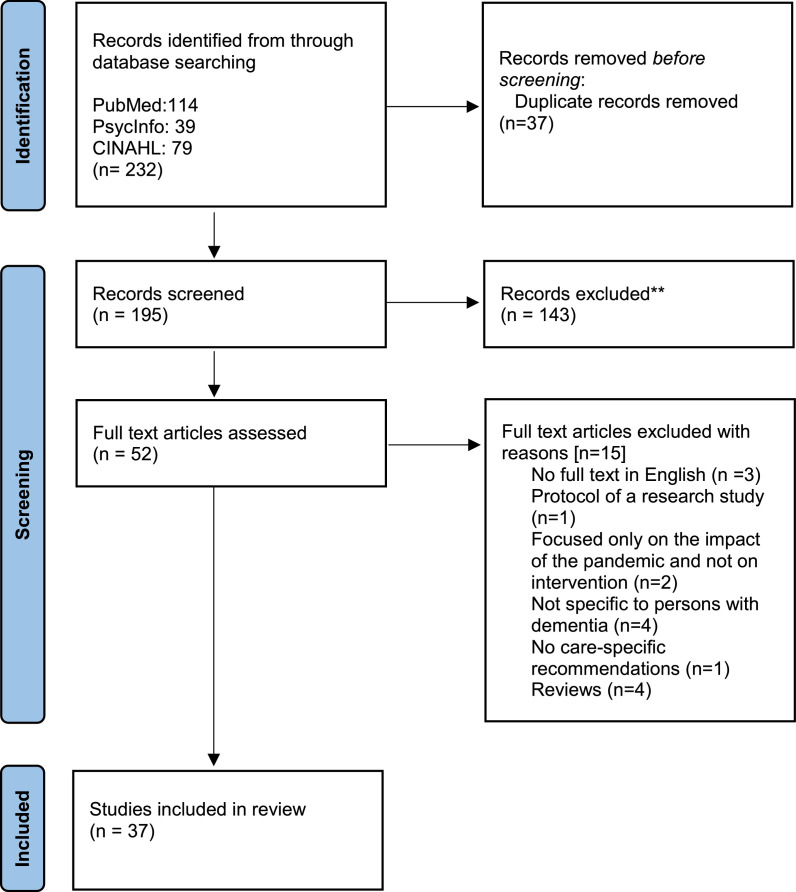
PRISMA diagram

Figure 1 provides an overview of the study selection process. Among the articles/studies included, there were 14 articles [6,11,[23], [24], [25], [26], [27], [28], [29], [30], [31], [32], [33], [34]], 14 primary research studies [35], [36], [37], [38], [39], [40], [41], [42], [43], [44], [45], [46], [47], [48], 4 perspective articles [4,[49], [50], [51]], 2 commentaries [52,53], 2 guidelines/recommendations [54,55] and 1 brief report [56]. These articles/studies were from (based on first author/location of research study) Canada [6,32,34,51,56], Italy [23,28,37,43,47,49], United States [26,27,29,33,35,39,41,42,52], United Kingdom [12,24,30,31,40,45,46,48], India [4,44], Turkey [25], Spain [36], Brazil [50], Singapore [53], Australia [54], and Poland [55].

For the search conducted on vaccinations, we identified a total of 81 records: 64 records from PubMed, 7 from PsychINFO and 10 from CINAHL. After removal of duplicates, 73 records were screened using their title and abstract, with 63/73 excluded after screening. We then assessed 10 full-text articles, with 5 articles [57,58,59,60,61] finally included in the review. Reasons for exclusion included: not specific to persons with dementia [N=2], court case commentary [N=1], not referring to safety of vaccinations [N=1] and reviews [N=1]. In addition, one article [62] was also included after searching Google Scholar. These were prospective cohort studies [57,62], a multi-centre longitudinal cohort study [58], case report [59] and a retrospective cohort study [60,61].

On reviewing these studies, several forms of interventions were identified to support persons with dementia and their families. Studies were grouped into four main themes that emerged: infection prevention, medical care, functional rehabilitation and care for persons with dementia, and caregiver support. These main themes were further divided into different sub-themes, which are summarized in Table 1, Table 2, Table 3, Table 4. Research studies and guidelines were suggested for patients residing in their homes or in residential settings. Countries across the world, from both high-income and low and middle-income reported their efforts.

**Table 1:** Guidelines for infection prevention.

Sl. No	Areas addressed	Description	Community Dwelling/Home, Hospital or Care Setting	Country (first author based)/Location	Type of publications
1	Curtailing movement to reduce risks of infections	To reduce risks of infection in care homes: explain social distancing, engage residents in stimulating activities in their own rooms, zone residents zone or transfer residents to different accommodations [24].	Care Setting	United Kingdom	Article
		Physical restrictions such as bed rails, tagged bracelets, door alarms [24] or a geriatric recliner with a pelvic holder (for short periods only) may be considered [34].	Care Setting	United Kingdom and Canada	Articles
		Medications should be used as a last resort [24,34,54]	Care and Hospital Setting	United Kingdom, Canada and Australia	Articles, guidelines
2	Provide reminders to persons with dementia on infection prevention and PPE	Remind persons with dementia on infection prevention measures such as wearing masks, social distancing and hand washing through signs/posters/photo instructions [29,53,55].	Community Dwelling and Care Setting	Poland, United States, Singapore	Expert recommendations, articles, commentaries
3	Design of Care Environment	Having a “small scale cluster design” in long term facilities such as care homes can facilitate the isolation of COVID-19 resident in single rooms and reduce spread of infection [32].	Care Setting	Romania	Research article
4	Limit social interactions	Restrict visitors [55] and ensure gatherings are done outdoors with social distancing [29].	Community Dwelling and Care Setting	Poland and United States	Expert recommendations, article
5	Education and training caregivers on infection prevention	Educate caregiver and staff on PPE and infection prevention [12,54] and incontinence management [33].	Hospital, Home and Care Setting	Australia, United Kingdom and United States	Guidelines, articles.
		They should model masks in order to normalize wearing of masks [29].	Community Dwelling	United States	Article

### 3.1 Infection prevention

Review of literature revealed several recommended measures to reduce risks of infection and maintain continuity of medical care. A crucial need for caregivers to provide continuous reminders to persons with dementia to maintain hygiene standards and reduce risks of infection through hand washing, wearing masks and social distancing were highlighted in the majority of studies [29,53]. Using visible prompts such as photos [55], signs or posters with large fonts in living areas were suggested in order to encourage such hygienic practices [29,53]. Additionally, having caregivers model masks was also recommended as measures to help to normalise the act for persons with dementia [29]. To further reduce infection risks, it was generally advised that caregivers restrict visitors [55] and that if family gatherings are held, they be conducted outdoors with adequate social distancing [29].

In long-term care settings, additional measures needed to be taken to reduce risks of infection such as training for caregivers to implement a strong infection prevention management plan [33]. Strategies to curtail wandering were suggested, and included explaining social distancing, zoning residents according to exposure or moving residents to an alternative accommodation if necessary [24]. Physical restrictions such as bed rails, door alarms, tagged bracelets and a geriatric recliner with a pelvic holder for short durations were also considered [24,34]. Pharmacological interventions such as sedatives however were perceived as a last resort option, as they could increase the risks of injury or falls in elderly [24,34,54]. Considering such difficulties in isolating residents and restricting wandering in large care home settings, future long-term care models should be based on smaller scale (10-12 residents per home) cluster like designs, as they allow for residents to isolate in a single bedroom, reduce risk of falls and minimize use of physical restrictions or medications [32]. A brief overview of the recommendations discussed to prevent infection among persons with dementia are provided in Table 1.

### 3.2 Medical care

Strategies were also developed to diminish disruptions in medical care provision. Telemedicine during the COVID-19 pandemic became the primary and effective way to deliver medical care to vulnerable persons with dementia without exposing them to infection risks [4,26,27,30,35,37,42,52,55]. This included availability of helpline numbers [4], tele and video consultations that allow an audio-visual interaction of the patient with their primary care physician, geriatrician, psychologist or occupational therapist [50,55]. In order to maintain clinical stability and monitoring for cognitive abilities and COVID symptoms, digital technology [37,50] was advocated. Due to difficulties in making a diagnosis via teleconsultations; it was recommended that initial consults be deferred or conducted in-person as often as possible, with follow-ups then carried out remotely [6]. In addition, direct delivery of medications to homes, obtaining prescriptions for a longer periods of time and regular monitoring of medications by pharmacy and non-pharmacy staff were suggested to ensure medication compliance and safety [28,29,40,55]. The need to identify precipitating factors for delirium such as sleep deprivation, pain, constipation and urinary retention and institute prompt treatment was emphasised [4].

In addition, a few studies have examined the effectiveness and impact of vaccinations on persons with dementia. A study conducted across five long-term care facilities in Spain found that the Pfizer (mRNA) vaccine was safe in older adults [the sample included those with mild to severe dementia] and that it provided immunogenicity irrespective of old age, fragility, and co-morbidities [58]. Another study examining the roll-out of the first dose of the Oxford-Astrazeneca and Pfizer COVID-19 vaccine in Scotland found that the mass roll out of the first doses of vaccinations were associated with considerable reductions in hospital admissions [62]. However, few studies have highlighted some concerns including delirium in a person with dementia after receiving CoronaVac, but this resolved quickly when treated [59]; severe COVID-19 infection that may still occur in partially or completely vaccinated individuals [with either Pfizer or Oxford-AstraZeneca] with co-morbidities including persons with dementia [57,60] and mortality post-vaccination in long-term care [LTC] facilities [61]. Although Lv et al. [61] further highlights that the mortality rate post-vaccination among LTC residents is relatively low.

A brief overview of the recommendations that can be followed to support medical care for persons with dementia are provided in Table 2.

**Table 2:** Guidelines for managing medical care.

Sl. No	Areas addressed	Description	Community Dwelling/Home, Hospital or Care Setting	Country (first author based)/Location	Type of publications
1	Telemedicine	Telehealth consultations can be used to provide care and monitor patients remotely [5,7,26,27,30,35,37,40,42,52,55].	Community, Dwelling, Care Setting, Hospital	United States, Italy, India, United Kingdom, Italy, Poland, Brazil, Canada	Primary research, perspective articles, commentary, expert recommendations
2	Medicine management	Have medications delivered to homes [28,40]. Prescriptions can be sent directly to pharmacies via email [37]. Obtain prescriptions for long periods [29,55]	None specified [28],Community Dwelling, Care Setting	United KingdomItaly, Poland, United States	Primary research, articlesexpert recommendations
3	Incontinence management	Having a good incontinence and infection prevention management plan which includes usage of good products and training of staff is crucial [33].	Care Setting	United States	Article
4	Treating delirium	Guidance to avoid and promptly treat precipitating factors for delirium [12].	Community Dwelling, Care Setting	United Kingdom	Article
5	Vaccinations	Vaccinations have been found to be safe in elderly and people with co-morbidities.* few risks have also been identified by other studies and a risk-benefit analysis must be done per person with dementia.	Long-term care, Hospital-based	Spain, Scotland	Primary research, prospective cohort

### 3.3 Functional rehabilitation and care for persons with dementia

Review of articles revealed that rehabilitation and care for persons with dementia was provided in the following major areas: multidisciplinary care, cognitive stimulation, reducing behavioral and psychological symptoms, help for activities of daily living (ADLs), environmental modifications, physical exercise, overcoming social isolation, general wellbeing and safety, and advanced care. The recommendations for functional rehabilitation and care for persons with dementia is summarized in Table 3.

**Table 3:** Guidelines for functional rehabilitation and multidisciplinary care.

Sl. No	Areas addressed	Description	Community Dwelling/Home, Hospital or Care Setting	Country (first author based) /Location of study	Type of publications
1	Cognitive stimulation	Modifications to cognitive stimulation programs to include leisure activities that can be done independently by PWD [25,34,54], smaller group activities; purposeful indoor activities [28,55] to retain skills and functional independence.	Care Setting, Community Dwelling, Hospitals	Canada, Turkey, Australia,Poland, Italy	Articles, guidelines, expert recommendations
		Online cognitive stimulation techniques [23,34]; Smartphones [28]; Virtual community resources, TV assisted cognitively stimulating and socially engaging activities and rehabilitation [36,53].	Care Setting, None Specified [28],Community Dwelling,	Canada, Italy, Singapore, Spain	Articles, primary research, commentary
2	Reducing behavioral disturbances	Relocating rooms towards open spaces, engagement in purposeful activities, sedatives, physical restraints, door locks, door alarms, tagged bracelets [24].	Care Setting	United Kingdom	Article
		Frequent monitoring and screening for delirium and behavioral changes [12,53].	Care Setting, Community Dwelling	United Kingdom, Singapore	Article, commentary
		Encouragement to exercise or walk close to homes [51,55].	Care Setting, Hospital, Community Dwelling	Canada, Poland	Perspective article, expert recommendations
		Reduction of exposure to news programs on TV or radio, media reports on COVID [55].	Care Setting, Community Dwelling	Poland	Expert recommendations
		Appropriate medication usage, telephone based reality orientation therapy (T-ROT) [47] and caregiver guidance [47].	Community Dwelling, None Specified [50]	Italy, Brazil	Primary research, perspective article
3	Help for activities of daily living	Electronic reminders [23].	None Specified [23]	Italy	Article
		TV assistance for daily living activities [36].	Community Dwelling	Spain	Primary research
		Food, grocery, drug deliveries and other essentials from local markets and pharmacists [35,28].	Community Dwelling, None Specified [28]	United States, Italy	Primary research, article
		Support for incontinence management [33].	Care Setting	United States	Article
4	Multidisciplinary care	Helpline numbers, Telemedicine to include teleconsultations and specifically video consultations with psychologists or occupational therapists [5,50,55].	Community Dwelling, Care Setting,	Poland, United Kingdom,Brazil, India, United States	Expert recommendations, primary research, article, perspective articles
		Monitoring for cognitive abilities and COVID-19 symptoms [37].	Community Dwelling	Italy	Primary research
		Initial in-person followed by remote online consultations [7].	Community Dwelling, Care Setting	Canada	Article
		Improving communication to overcome the barrier provided by masks and face-shields [5].	Community Dwelling	India	Perspective article
		Collaborations between primary and secondary care for transitional care, multidisciplinary and holistic long term care [12].	Community Dwelling, Care Setting.	United Kingdom	Article
		Partnering with governmental initiatives for community dementia care and support [5].	Community Dwelling	India	Perspective article
5	Environmental modification	Orienting PWD to their physical environment [34].	Care Setting	Canada	Article
		Technology enabled interactions with family members and health care staff [56].	Care Setting	Canada	Brief report
6	Physical exercise	Daily exercises and walking indoors and in parks [51], [52], [53]	Community Dwelling, Care Setting	Singapore, Canada, United States	Commentaries, perspective article
		Video-call based exercise programmes [45].	Community Dwelling	United Kingdom	Primary research
7	Overcoming social isolation	Frequent phone calls and emails [39,51], TV and telehealth technologies, videoconferencing, tablet-based applications, game consoles, assistive-technologies using virtual reality to enhance social interaction among nursing home residents and family [27,28,49,56].	Care Setting, Hospital, Community Dwelling	United States, Canada, Italy	Primary research, perspective articles, article, brief report
		Window visits [56].	Care Setting	Canada	Brief report
		Telepresence robots to enable caregivers and loved ones to render reassurance and comfort [53].	Community Dwelling, Care Setting	Singapore	Commentary
		“link up’ of multiple care homes through video calls using Skype/FaceTime/ Zoom to stay connected [46].	Care Setting	United Kingdom	Primary research
8	General wellbeing and safety	Relaxation or meditation exercises provided via electronic media; quality of life, physical and mental wellbeing assessment using telemedicine via videoconferencing apps [38].	Home and care setting	Hong Kong	Primary research
		Demonstration of resilience by adult day care centers (ADCs) that were able to remain operational by accessing economic, social and political capital [41].	Care Setting	United States	Primary research
		Strategies to navigate re-entry of PWD into society safely after lifting of lockdown [29].	Community Dwelling	United States	Article
		Video telehealth allow to address physical or safety concerns [26].	None Specified	United States	Article
9	Advanced care	Advanced care planning with family members and dealing with bereavement [5,26,31,51,53].	None Specified [26],Care Setting, Community Dwelling, Hospital	United States, United Kingdom, Singapore, Canada, India	Articles, commentary, perspective articles
		Digital programs for end-of-life planning between nursing home staff and families [12,27,31].	Care Setting, Community Dwelling	United States, United Kingdom	Articles
		Grief counselling and supportive therapy for PWD experiencing death of peer residents due to COVID-19 [51].	Care Setting, Community Dwelling, Hospital	Canada	Perspective article

Dementia care is multidisciplinary in nature and during the pandemic, consultations with psychologists, occupational therapists and speech pathologists was supported through use of technology [4,50,55]. The importance of maintaining communication with persons with dementia during the pandemic was stressed and the use of audio-visual aids, voice modulation and non-verbal communication were advised to overcome the barriers caused by masks and face-shields [4].

Cognitive stimulation therapy for dementia includes a range of activities that can be individualized and be applied under home conditions [63]. These activities have been found to have a positive impact on cognition, quality of life (QoL), and general well-being of persons with dementia and caregivers [64]. During the COVID-19 pandemic, anticipating a major interruption of these activities, several expert groups and research studies focused on adapting and modifying existing cognitive stimulation therapy programs for persons with dementia who were in isolation or lacked professional face-to-face support. Some of the simpler yet effective modifications were to provide activities that could be undertaken individually, without much supervision such as coloring pages and math worksheets [34]; knitting, painting and cleaning [25]. Activities in small groups were also encouraged [54], as were purposeful indoor activities to retain skills and functional independence such as sorting out old photographs, collecting newspaper clippings, listening to old songs, cleaning, organising and cooking [28,55]. These activities were found be particularly relevant during COVID-19 restrictions and were associated with improved cognitive performance among elderly.

To compensate for the interruption of face-to face interactions, online cognitive stimulating and socially engaging activities were implemented across many groups supporting persons with dementia. Leisure interests such as playing music and movies on tablets, and internet-based games [23,34]; smartphones, game consoles, and TV assisted activities to keep individuals engaged and stimulated [28,36] and engagement in telecognitive stimulation therapy or rehabilitation programmes [53] found acceptance during the COVID-19 pandemic.

Various interventions were found helpful for management of behavioural and psychological symptoms in persons with dementia, and included environmental modifications, physical exercise and psychological treatment [51,65,66]. Experts from multiple disciplines recommended engagement in purposeful activities to reduce wandering [24]. Screening and frequent monitoring for delirium, emotional and behavioural changes due to social isolation, and reducing exposure to news programs on TV or radio or media reports on COVID-19 to reduce stress were recommended [11,53,55]. The use of door locks, door alarms, tagged bracelets, and if needed judicious use of sedatives and physical restraints were advised to reduce wandering [24,51]. Technology based solutions such as telephone based reality orientation therapy (T-ROT) combined with emotional support helped manage behavioural and psychological symptoms of patients with major neurocognitive disorders who were forced to isolate during the pandemic [47].

Many care-settings worldwide recommended environmental modifications especially during the pandemic such as posting signs in rooms for orientation [34]; wall-mounted screens with easy-to-use visuals where residents can press an image of their family member to make a video call, and loud-speakers or audio that connects directly to hearing aids were some of the innovations developed [56].

Regular physical activity and a healthy lifestyle has been found to delay the progression of dementia, improve cognitive functioning, reduce frailty, and lower the risk of depression and other behavioral and psychological symptoms [67], [68], [69], [70], [71]. Amidst the COVID-19, both primary research studies and expert guidelines encouraged walking/daily exercises indoors or in the nearby parks [51], [52], [53]. Di Lorita et al., [45] implemented the promoting activity, independence and stability in early dementia (PrAISED) which is a home-based, face-to-face, individually tailored exercise program for PWD via remote interviews (telephone/video calls). PrAISED provided effective strategies to deliver rehabilitation remotely in situations with social distancing during the pandemic.

Preserving and improving the activities of daily living (ADLs) is a foremost patient related outcome, and this was also emphasised during the COVID-19 pandemic. Some of the interventions implemented to maintain ADLs were the use of electronic reminders [23,36], environmental modifications [63] and special delivery arrangements for food, drugs, and other essential items from supermarkets, local grocers, and pharmacies [28].

To prevent persons with dementia from being socially isolated, various experts provided guidelines developed through practice and research to enhance social interaction among nursing home residents and family. Window visits to family members, i.e., setting up large windows where persons with dementia and family members interact with each other were offered by residential care homes [56]. Telehealth technologies were widely used such as videoconferencing, tablet-based applications [27], assistive technologies using virtual reality [49], and frequent phone calls and emails [39,51]. Interestingly, engaging family in technology-based games was advised to reduce boredom and isolation of persons with dementia [28,56]. Furthermore, multiple care homes “linked up” through video calls using Skype or FaceTime or Zoom to stay connected [46]. Telepresence robots were also developed to provide comfort and support from caregivers [53].

Several collaborative approaches were initiated to support dementia care in the community such as partnerships with governmental initiatives for community dementia care and support via telephonic, video and social media platforms [4]. Collaborations between primary and secondary care for transitional care, multidisciplinary and holistic long term care for prompt provision of psychosocial support were also developed [11].

The general wellbeing and safety during COVID-19 pandemic was addressed through tele-assessments [video and phone consultations] for quality of life, physical and mental wellbeing and by providing relaxation and meditative exercises using electronic media [26,38]. Additionally, adult day care centers (ADCs) demonstrated resilience and were able to remain operational by accessing economic capital in the form of grants; social capital by developing collaborative relationships with caregivers and political capital by obtaining health directives for reopening of ADCs [41].Strategies were also developed to navigate re-entry of persons with dementia into society with the implementation of recreational activities that are vehicle friendly (movies, drive through zoos, etc.), and hosting family gatherings in safe spaces preferably open spaces [29].

Additionally, to support terminal illness of persons with dementia during the pandemic, the usage of electronic and audiovisual programs were advised for end-of-life planning, dealing with bereavement, and information sharing between nursing homes and families and to provide a useful avenue of support [4,11,26,27,31,51,53]. Grief counselling or supportive therapy was also provided to persons with dementia when other residents with dementia experienced death [51].

### 3.4 Caregiver support

A number of recommendations have been provided to reduce caregiver stress and support caregivers during the COVID-19 pandemic. Reaching out to caregivers through various mobile and web-based platforms such as text messaging, chat forums or video recordings can aid in ensuring that specific needs of caregivers are brought to the attention of health professionals [4,23,50]. Physicians and allied health professionals (e.g., occupational therapists, physiotherapists etc.) can also be contacted through teleconsultations [50] and through local helpline numbers available in different countries [4]. Through such remote measures, caregivers can receive training and psycho-educational programs, thereby equipping them with essential knowledge during the pandemic to address new challenges [23,50]. Such family support interventions were found to reduce caregiver burden during the COVID-19 pandemic [43]. In addition, caregivers should also be provided specific strategies to engage their relative with dementia (e.g., driving PWD to familiar places, going to outdoor green spaces before crowds pick up), while continuing to protect their loved one and themselves from infection risks [29].

Besides addressing the medical and psychosocial needs of caregivers, the crucial need for other essential services such as food and medication was also recognised. Deliveries can be provided by local markets and pharmacies [28,35]. Foreseeing that caregivers are also likely to be struggling with loss of income or employment during the pandemic; provision of social security and remuneration to family caregivers was recommended [4]. The state of Kerala in India, for example, provides Rs. 600 per month to female unpaid caregivers of individuals with mental or physical illness; a similar model can also be applied to family caregivers of persons with dementia [4]. The recommendations for caregiver support are presented in Table 4.

**Table 4:** Guidelines for caregiver support.

Sl. No	Areas addressed	Description	Community Dwelling/Home, Hospital or Care Setting	Country (first author based) /Location of study	Type of Publications
1	Training for cognitively stimulating activities	To improve knowledge and competence for caregiving; information, teleconsultation and web-based training on CST strategies and techniques [23,50].	None Specified	Italy, Brazil	Article, perspective article
2	Addressing caregiver stress and burden	To reduce caregiver psychological burden through counselling, mobile text messaging, video recording and chat forums [5,23,50].	None Specified [23,50], Community Dwelling	Italy, India, Brazil	Article, perspective articles.
3	Support for basic needs	Food, grocery, drug deliveries and other essentials from local markets and pharmacists [28,35].	Community Dwelling, None Specified [28].	United States, Italy	Primary research, article
4	Medical and psychosocial care	Consultations with physicians, psychologists or occupational therapists through helpline numbers [5], teleconsultations [50].	Community Dwelling, None Specified [50]	India, Brazil	Perspective articles
		Family support through training on disease management and providing knowledge on local public care services [43].	Community Dwelling	Italy	Primary research
		Guidance for empowering caregivers to advocate for their loved ones (COP^2^E) [29].	Community Dwelling	United States	Article
5	Financial support	Provision of social security and remuneration of informal caregivers [5].	Community Dwelling	India	Perspective article
6	Support for health professionals and administrators	Online learning systems and resources to mitigate workforce stress and improve quality of care [27,33].	Care setting	United States	Guidelines
		Sensitising physicians for use of telemedicine [44].	Community Dwelling	India	
		Interim guidance for health professionals and administrators providing hospital care to adults with cognitive impairment, including appropriate medication use [54].	Hospital	Australia	

## 4 Summary and conclusions

A detailed review of literature of dementia care during the COVID-19 pandemic revealed that a wide range of helpful measures were developed towards support of persons with dementia and their caregivers. The main areas of care included measures to prevent infection specific to persons with dementia, medical care, functional rehabilitation and support for persons with dementia and caregiver support

As the COVID-19 pandemic spread quickly across the world from when the first cases were reported in China in December 2019, persons with dementia have been amongst the most vulnerable groups affected. This has raised concerns for dementia care as early as March 2020 [72]. There have been continued efforts from clinicians, dementia support organizations, professional medical bodies towards amending available care and support services for persons with dementia and their caregivers in addition to developing new care strategies [5,20,23,38,46,55]. This published evidence has provided guidance on not only delivering better care to persons with dementia amidst the COVID-19 pandemic, but also suggested recommendations on infection prevention and protection specific to persons with dementia as well as support for caregivers [24,28,29,34,54]. In addition, with the administration of COVID-19 vaccinations in 2021, few studies have evaluated safety as well as risks of hospital admission/mortality from COVID-19 after vaccinations in elderly and persons with dementia [57], [58], [59], [60], [61], [62]. The literature reviewed indicated that these efforts were put forth by both high-income and low-middle income countries including but not limited to Canada, Italy, Israel, United States, United Kingdom, Australia, Spain, Singapore, India, Turkey, and Brazil.

While most published studies were in the form of guidelines and recommendations, there were several of primary research studies that were conducted [35], [36], [37], [38], [39], [40], [41], [42], [43], [44], [45], [46], [47], [48]. The majority focused on telemedicine; demonstrating it to be a plausible way to effectively monitor persons with dementia [37,38,42,48] and to conduct rehabilitation activities remotely [36,45,47]. In addition, few studies identified coping strategies adopted by family members of persons with dementia [35], determined the effectiveness of family support interventions in reducing caregiver burden [43] and revealed the positive impact of maintaining social connections using remote methods on persons with dementia or cognitive impairment living in long-term care settings during the pandemic period [39,46]. Three of the 14 research studies spoke to expert groups such as pharmacists [40], general physicians [44] and assisted living community staff [41], to better understand how to best support persons with dementia and their families during the pandemic period.

Infection prevention guidelines and recommendations, medical care, functional rehabilitation, multidisciplinary care for persons with dementia amidst the COVID-19 pandemic was the focus of most intervention guidelines and research studies [24,28,29,33,36,45,53,55]. A positive response that emerged from the pandemic was the development of online cognitive stimulation therapy. Most of the published literature provides recommendations for using online cognitive interventions, however, there was one study that demonstrated that participants with mild dementia and mild cognitive impairment (MCI) benefitted when provided with television based assistive integrated cognitive stimulation service (TV-AssistDEM) [36]. It is further crucial to train caregivers to manage care for persons with dementia during periods of emergency. Additionally, mechanisms to provide social security and remunerate family caregivers be developed particularly in lower-middle-income countries (LMICs) [5] in order reduce the impact of future health emergencies on vulnerable populations.

Technology provides new opportunities for assessment, management, and follow-up care for persons with dementia to maintain functional independence and safety and to improve quality and access to care [57,67]. Telemedicine, which involves audio-visual interaction between the physician and persons with dementia and caregiver, has been identified as an effective way to make a diagnosis, maintain clinical stability of patients and monitor persons with dementia for cognitive impairment and COVID symptoms remotely, without exposing them to infection risks, and provide caregiver support and training [4,23,26,27,30,35,37,40,42,48,50,52,55]. Technology driven services can potentially be a promising solution for PWD residing in various remote geographical locations or rural areas that are underserved, to provide continued therapy and support.

While COVID-19 pandemic has accelerated the use of telemedicine and telerehabilitation for consultations and management of persons with dementia, there are some concerns that are encountered while using such modern technology: (i) the quality of internet connection in certain geographical locations and socio-economic groups may not be good [38]; (ii) the presence of cognitive and sensory deficits may make it difficult for persons with dementia to use the technology and their caregivers might not be very digitally literate [26,45,50]; (iii) many persons with dementia and their relatives may not possess the digital technology/gadgets required [46]; (iv) assessments during teleconsultations at times might be difficult as persons with dementia and their caregivers might not be able to follow the instructions provided to download, answer and perform the tasks [50]; and (v) telemedicine limits the ability of physicians to perform both physical and neurological examinations [37] and physicians need to discuss this limitation with persons with dementia and their caregivers before giving any advice [5].

In conclusion, while the COVID-19 pandemic has exposed the vulnerabilities of persons with dementia and their caregivers, it has also revealed the resilience and commitment of the dementia community to reframe existing services and provide care during periods of emergency. The multidisciplinary requirement of dementia care was recreated through a variety of services that encompassed medical, non-pharmacological and psychosocial care strategies. The ongoing pandemic emphasises the need to continue these efforts and build evidence for their effectiveness, and ensure their implementation across global communities.

## Funding

This research did not receive any specific grant from funding agencies in the public, commercial, or not-for-profit sectors.

## Appendix Supplementary materials

Image, application 1

Image, application 2
