# Supplementary material for: Care for dementia patients and caregivers amid COVID-19 pandemic
Source: Cereb Circ Cogn Behav. 2022 Jan 18;3:100040. doi: 10.1016/j.cccb.2022.100040 (PMC8763414; doi:10.1016/j.cccb.2022.100040)
Supplement: Supplementary file 1 [file mmc1.docx]

**Appendix 1: Search Strategy**

**PubMed**

Concept 1:

(dementia [MeSH Terms] OR dementia [tiab] OR alzheimer’s disease [MeSH Terms] OR alzheimer’s disease [tiab] OR vascular dementia [MeSH Terms] OR vascular dementia [tiab] OR frontotemporal dementia [MeSH Terms] OR frontotemporal dementia [tiab] OR neurodegenerative disorders [MeSH Terms] OR cognitive impairment [tiab]).

Concept 2:

(coronavirus [MeSH Terms] OR coronavirus*[tiab] OR covid*[tiab] OR covid-19* [tiab] OR “sars coronavirus 2” [tiab] OR “sars cov 2” [tiab] OR sars-cov-2 [tiab] OR “severe acute respiratory syndrome coronavirus 2” [tiab] OR “severe acute respiratory syndrome cov*” [tiab] OR ncov* [tiab] OR wuhan [tiab] OR pandemic [tiab] OR lockdown [tiab] OR outbreak [tiab] OR quarantine [tiab])

Concept 3:

(caregivers [MeSH Terms] OR caregivers [tiab] OR caregiving [tiab] OR caring [tiab] OR family caregivers [MeSH Terms] OR family caregivers [tiab] OR informal care [tiab] OR community based care [tiab] OR community-based care [tiab] OR community-dwelling* [tiab] OR long term care [Mesh Terms] OR long term care [tiab] OR long-term care OR aged care [tiab] OR home based care [tiab] OR home-based care [tiab] OR home care [tiab] OR elder care [tiab] OR skilled nursing facility [MeSH Terms] OR residential facilities [MeSH Terms] OR housing for the elderly [MeSH Terms] OR nursing homes [MeSH Terms] OR palliative care [MeSH Terms] OR physical care [tiab])

Concept 4:

(medical care [tiab] OR telemedicine [MeSH Terms] OR telemedicine [tiab] OR telehealth [tiab] OR digital technolog* [tiab] OR electronic health records [MesH] OR patient record* [tiab] OR app [tiab] OR video calls [tiab] OR phone calls [tiab] OR pharmacological* [tiab] OR infection prevention [tiab] OR infection control [tiab] OR social interaction [MeSH Terms] OR social interaction [tiab] OR cognitive behavior therapies [MeSH terms] OR cognitive stimulating* [tiab] OR cognitive stimulation* [tiab] OR functional rehabilitation [tiab] OR rehabilitation [tiab] OR exercise [tiab] OR physical activities [MeSH Terms] OR physical activities [tiab] OR cognitive enhancement [tiab] OR support group* [MeSH Terms] OR support group* [tiab] OR support [tiab] OR counseling [MeSH terms] OR counseling [tiab] OR care coordination*[tiab] OR health measures [tiab] OR web-based interventions [tiab] OR web based interventions [tiab] OR at-home therapy [tiab] OR at home therapy [tiab] OR advocacy [tiab])

**PsychINFO**

Concept 1:

(exp dementia/ OR dementia.tw. OR exp Alzheimer’s Disease/ OR alzheimer’s disease.tw OR exp vascular dementia/ OR vascular dementia.tw OR frontotemporal dementia.tw OR exp neurodegenerative diseases/ OR cognitive impairment.tw)

Concept 2:

(exp coronavirus/ OR coronavirus.tw OR covid*.tw OR covid-19*.tw OR sars coronavirus 2.tw OR sars cov 2.tw OR sars-cov-2.tw OR severe acute respiratory syndrome coronavirus 2.tw OR severe acute respiratory syndrome cov*.tw OR ncov*.tw OR wuhan.tw OR pandemic.tw OR lockdown.tw OR outbreak.tw OR quarantine.tw)

Concept 3:

(exp caregivers/ OR caregivers.tw OR caregiving. tw OR caring.tw OR family caregivers.tw OR informal care.tw OR community based care.tw OR community-based care. tw OR community-dwelling* OR exp long term care/ OR long term care.tw OR long-term care.tw OR aged care.tw OR home based care.tw OR home-based care.tw OR home care.tw OR elder care.tw OR exp residential care institutions/ OR exp nursing homes/ OR exp palliative care/ OR physical care.tw)

Concept 4:

(medical care.tw OR exp telemedicine/ OR telemedicine.tw OR telehealth.tw OR digital technolog*.tw OR exp electronic health records/ OR patient record*.tw OR app.tw OR video calls.tw OR at OR pharmacological*.tw OR infection prevention.tw OR infection control.tw OR exp social interaction/ OR social interaction.tw OR exp cognitive behavior therapy/ OR cognitive stimulating*.tw OR cognitive stimulation*.tw OR functional rehabilitation.tw OR rehabilitation.tw OR exercise.tw OR exp physical activity/ OR physical activities.tw OR cognitive enhancement.tw OR exp support groups/ OR support group*.tw OR support.tw OR exp counseling/ OR counseling.tw OR care coordination*.tw OR health measures.tw OR web-based interventions.tw OR web based interventions.tw OR at-home therapy.tw OR at home therapy.tw OR advocacy.tw)

**CINAHL**

Concept 1:

((MH “Dementia+”) OR (TI dementia OR AB dementia) OR (MH “Alzheimer’s Disease+”) OR (TI Alzheimer’s disease OR AB Alzheimer’s Disease) OR (MH “Vascular Dementia+”) OR (TI vascular dementia OR AB vascular dementia) OR (MH “frontotemporal dementia+”) OR (TI frontotemporal dementia OR AB frontotemporal dementia) OR (MH “neurodegenerative disorders+”) OR (TI cognitive impairment OR AB cognitive impairment))

Concept 2:

(( MH “Coronavirus+”) OR (TI coronavirus OR AB coronavirus) OR (TI covid* OR AB covid*) OR (TI covid-19* OR AB covid-19*) OR (TI sars coronavirus 2 OR AB sars coronavirus 2) OR ( TI sars cov 2 OR AB sars COV 2) OR (TI sars-cov-2 OR AB sars-cov-2) OR (TI severe acute respiratory syndrome coronavirus 2 OR AB severe acute respiratory syndrome coronavirus 2) OR (TI severe acute respiratory syndrome cov* OR AB severe acute respiratory syndrome cov*) OR (TI ncov* OR AB ncov*) OR (TI wuhan OR AB wuhan) OR (TI lockdown OR AB lockdown) OR (TI pandemic OR AB pandemic) OR (TI outbreak OR AB outbreak) OR (TI quarantine OR AB quarantine))

Concept 3:

((MH “caregivers or family members+”) OR (TI caregivers OR AB caregivers) OR (MH “caregiving+”) OR (TI caring OR AB caring) OR (TI family caregivers OR AB family caregivers) OR (TI informal care OR AB informal care) OR (TI community based care OR AB community based care) OR (TI community-based care OR AB community-based care) OR (MH “long term care+”) OR (TI long term care OR AB long term care) OR (TI long-term care OR AB long-term care) OR (TI aged care OR AB aged care) OR (TI home care OR AB home care) OR (TI home based care OR AB home based care) OR (TI home-based care OR AB home-based care) OR (TI elder care or AB elder care) OR (MH “residential facilities+”) OR (MH “housing for the elderly+”) OR (MH “nursing home+”) OR (MH “palliative care+”) OR (TI physical care OR AB physical care))

Concept 4:

((TI medical care OR AB medical care) OR (MH “Telemedicine+”) OR (TI telemedicine or AB telemedicine) OR (MH “Telehealth+”) OR (TI telehealth OR AB telehealth) OR (TI digital technolog* OR AB digital technolog*) OR (MH “electronic health records+”) OR (TI patient record* OR AB patient record*) OR (TI app OR AB app) OR (TI video calls OR AB video calls) OR (TI phone calls OR AB phone calls) OR (TI pharmacological* OR AB pharmacological*) OR (TI infection prevention OR AB infection prevention) OR (TI infection control OR AB infection control) OR (MH “social interaction+”) OR (TI social interaction OR AB social interaction) OR ( MH “cognitive behavioral therapy+”) OR (TI cognitive stimulating* OR AB cognitive stimulating*) OR (TI cognitive stimulation* OR AB cognitive stimulation*) OR (TI functional rehabilitation OR AB functional rehabilitation) OR (TI rehabilitation OR AB rehabilitation) OR (TI exercise OR AB exercise) OR (MH “physical activity+”) OR (TI physical activities OR AB physical activities) OR (TI cognitive enhancement OR AB cognitive enhancement) OR (MH “support groups+”) OR (TI support groups OR AB support groups) OR (TI support OR AB support) OR (MH “counseling+”) OR (TI counseling OR AB counseling) OR (TI care coordination* OR AB care coordination*) OR (TI health measures OR AB health measures) OR (TI web-based interventions OR AB web-based interventions) OR (TI web based interventions OR AB web based interventions) OR (TI at-home therapy OR AB at-home therapy) OR (TI at home therapy OR AB at home therapy) OR (TI advocacy or AB advocacy)).
