# Supplementary material for: Care for dementia patients and caregivers amid COVID-19 pandemic
Source: Cereb Circ Cogn Behav. 2022 Jan 18;3:100040. doi: 10.1016/j.cccb.2022.100040 (PMC8763414; doi:10.1016/j.cccb.2022.100040)
Supplement: Supplementary file 2 [file mmc2.docx]

**Appendix 2: Search Strategy Vaccinations**

**PubMed**

Concept 1:

(dementia [MeSH Terms] OR dementia [tiab] OR alzheimer’s disease [MeSH Terms] OR alzheimer’s disease [tiab] OR vascular dementia [MeSH Terms] OR vascular dementia [tiab] OR frontotemporal dementia [MeSH Terms] OR frontotemporal dementia [tiab] OR neurodegenerative disorders [MeSH Terms] OR cognitive impairment [tiab]).

Concept 2:

(coronavirus [MeSH Terms] OR coronavirus*[tiab] OR covid*[tiab] OR covid-19* [tiab])

Concept 3:

(vaccination[MeSH Terms] OR vaccination* [tiab] or vaccine* [tiab])

**PsychINFO**

Concept 1:

(exp dementia/ OR dementia.tw. OR exp Alzheimer’s Disease/ OR alzheimer’s disease.tw OR exp vascular dementia/ OR vascular dementia.tw OR frontotemporal dementia.tw OR exp neurodegenerative diseases/ OR cognitive impairment.tw)

Concept 2:

(exp coronavirus/ OR coronavirus.tw OR covid*.tw OR covid-19*.tw)

Concept 3:

(exp immunization/OR vaccine*.tw OR vaccination*.tw)

**CINAHL**

Concept 1

((MH “Dementia+”) OR (TI dementia OR AB dementia) OR (MH “Alzheimer’s Disease+”) OR (TI Alzheimer’s disease OR AB Alzheimer’s Disease) OR (MH “Vascular Dementia+”) OR (TI vascular dementia OR AB vascular dementia) OR (MH “frontotemporal dementia+”) OR (TI frontotemporal dementia OR AB frontotemporal dementia) OR (MH “neurodegenerative disorders+”) OR (TI cognitive impairment OR AB cognitive impairment))

Concept 2

(( MH “Coronavirus+”) OR (TI coronavirus OR AB coronavirus) OR (TI covid* OR AB covid*) OR (TI covid-19* OR AB covid-19*))

Concept 3

((MH “vaccines+”) OR (MH “immunization+”) (TI vaccine OR AB vaccine) OR (TI vaccination* OR AB vaccination*))
